# Supplementary material for: Bayesian refinement of protein structures and ensembles against SAXS data using molecular dynamics
Source: PLoS Comput Biol. 2017 Oct 18;13(10):e1005800. doi: 10.1371/journal.pcbi.1005800 (PMC5662244; doi:10.1371/journal.pcbi.1005800)
Supplement: S1 Archive — (BZ2) [file pcbi.1005800.s011.tar.bz2 › gromacs_4.62_bayesian_saxs_refi/share/html/online/g_current.html]

g\_current


| g\_current Main Table of Contents |

**VERSION 4.6  
Sat 19 Jan 2013**

---

### Description

g\_current is a tool for calculating the current autocorrelation function, the correlation
of the rotational and translational dipole moment of the system, and the resulting static
dielectric constant. To obtain a reasonable result, the index group has to be neutral.
Furthermore, the routine is capable of extracting the static conductivity from the current
autocorrelation function, if velocities are given. Additionally, an Einstein-Helfand fit
can be used to obtain the static conductivity.

The flag -caf is for the output of the current autocorrelation function and -mc writes the
correlation of the rotational and translational part of the dipole moment in the corresponding
file. However, this option is only available for trajectories containing velocities.
Options -sh and -tr are responsible for the averaging and integration of the
autocorrelation functions. Since averaging proceeds by shifting the starting point
through the trajectory, the shift can be modified with -sh to enable the choice of uncorrelated
starting points. Towards the end, statistical inaccuracy grows and integrating the
correlation function only yields reliable values until a certain point, depending on
the number of frames. The option -tr controls the region of the integral taken into account
for calculating the static dielectric constant.

Option -temp sets the temperature required for the computation of the static dielectric constant.

Option -eps controls the dielectric constant of the surrounding medium for simulations using
a Reaction Field or dipole corrections of the Ewald summation (-eps=0 corresponds to
tin-foil boundary conditions).

-[no]nojump unfolds the coordinates to allow free diffusion. This is required to get a continuous
translational dipole moment, required for the Einstein-Helfand fit. The results from the fit allow
the determination of the dielectric constant for system of charged molecules. However, it is also possible to extract
the dielectric constant from the fluctuations of the total dipole moment in folded coordinates. But this
option has to be used with care, since only very short time spans fulfill the approximation that the density
of the molecules is approximately constant and the averages are already converged. To be on the safe side,
the dielectric constant should be calculated with the help of the Einstein-Helfand method for
the translational part of the dielectric constant.

### Files

| option | filename | type | description |
| --- | --- | --- | --- |
| **-s** | topol.tpr | Input | Structure+mass(db): tpr tpb tpa gro g96 pdb |
| **-n** | index.ndx | Input, Opt. | Index file |
| **-f** | traj.xtc | Input | Trajectory: xtc trr trj gro g96 pdb cpt |
| **-o** | current.xvg | Output | xvgr/xmgr file |
| **-caf** | caf.xvg | Output, Opt. | xvgr/xmgr file |
| **-dsp** | dsp.xvg | Output | xvgr/xmgr file |
| **-md** | md.xvg | Output | xvgr/xmgr file |
| **-mj** | mj.xvg | Output | xvgr/xmgr file |
| **-mc** | mc.xvg | Output, Opt. | xvgr/xmgr file |

### Other options

| option | type | default | description |
| --- | --- | --- | --- |
| **-[no]h** | bool | no | Print help info and quit || **-[no]version** | bool | no | Print version info and quit || **-nice** | int | 0 | Set the nicelevel || **-b** | time | 0 | First frame (ps) to read from trajectory || **-e** | time | 0 | Last frame (ps) to read from trajectory || **-dt** | time | 0 | Only use frame when t MOD dt = first time (ps) || **-[no]w** | bool | no | View output .xvg, .xpm, .eps and .pdb files || **-xvg** | enum | xmgrace | xvg plot formatting: xmgrace, xmgr or none || **-sh** | int | 1000 | Shift of the frames for averaging the correlation functions and the mean-square displacement. || **-[no]nojump** | bool | yes | Removes jumps of atoms across the box. || **-eps** | real | 0 | Dielectric constant of the surrounding medium. The value zero corresponds to infinity (tin-foil boundary conditions). || **-bfit** | real | 100 | Begin of the fit of the straight line to the MSD of the translational fraction of the dipole moment. || **-efit** | real | 400 | End of the fit of the straight line to the MSD of the translational fraction of the dipole moment. || **-bvit** | real | 0.5 | Begin of the fit of the current autocorrelation function to a\*t^b. || **-evit** | real | 5 | End of the fit of the current autocorrelation function to a\*t^b. || **-tr** | real | 0.25 | Fraction of the trajectory taken into account for the integral. || **-temp** | real | 300 | Temperature for calculating epsilon. |

---

http://www.gromacs.org  
gromacs@gromacs.org
